# Electricity in the Diseases of Women

**Published:** 1889-04

**Authors:** 


BUFFALO MEDICAL AND SURGICAL JOURNAL

A MONTHLY REVIEW OF MEDICINE AND SURGERY.

EDITORS:

THOS. LOTHROP, M. D., - - - W. W. POTTER, M. D.

All communications, whether of a literary or business character, should be addressed to the editors: 284 FRANKLIN STREET, BUFFALO, N. Y.

### Editorial.

### *ELECTRICITY IN THE DISEASES OF WOMEN.*[1]

The volume before us is the first attempt at a systematic work on this subject which has been put before the profession, and furnishes the text for some observations thereon that we have had in contemplation for some time. It is, moreover, a subject that warrants the careful consideration of every physician, hence justifies a more detailed consideration than would be warranted in our review columns.

The want of such a treatise has long been felt by the general practitioner, and to this class of the profession it should be particularly valuable. That such has been the intention of the author, is very evident. To the specialist, this book will come more as a curiosity, as being a first attempt in this very seductive and important branch of gynecological work. Such an undertaking, to be of value to its intended audience, should begin by laying down as positive and absolute only such facts as are proven beyond cavil, and which are universally accepted. Secondly, it should exclude, or keep in the background, all such matter as may still be under dispute and trial. Thirdly, it should be written in a terse, brief, and clear manner, to the exclusion of all long and unnecessary discussions ; and, finally, should lay down, clearly and positively, some definite method of pro-

1. Electricity in the Diseases of Women, with special reference to the Application of Strong Currents. By G. Betton Massey, M. D., Physician to the Nervous Department of Howard Hospital ; Late Electro-Therapeutist to the Philadelphia Orthopedic Hospital and Infirmary for Nervous Diseases, etc., etc. 12mo, pp. 210. Philadelphia and London : F. A Davis, publisher. 1889. Price, $1.50 net.

cedure, with an equally definite armamentarium, accompanied by sufficiently concise details as to its use. The last two of these propositions have been most admirably carried out by the author, and are about what they should be. Let us see whether the first two have been lived up to in a becoming manner.

The work is divided pretty sharply into two general parts. The first, consisting of 101 pages, contains an introductory chapter, followed by chapters on the different electrical currents, together with a description of their actions and the methods of their application; and it also deals with the armamentarium. The second part is devoted to detailing the treatment of the individual diseases and proofs of the efficiency of the treatment.

It is in the first part that the author appears to good advantage; and he has here dealt with parts of his subject in a most sensible and thorough manner. He has simplified and made the whole subject most clear to those of the profession who have not the time to go deeply into technical or experimental studies and research. The three currents taken into consideration are the galvanic, faradic, and franklinic. For the use of the galvanic current, the articles considered absolutely necessary are a battery, a current-controller, a meter indicating at least 500 milliampères, and electrodes of various kinds. The battery preferred is a permanent one, consisting of about seventy cells of the Law telephone variety. Current-selectors are utterly condemned for any kind of battery, their place being taken by the current-controller. This controller is one of the author's own inventions, and seems to be an admirable one. The meter recommended is Flemming's, and is considered superior to those of foreign make, besides being cheaper.

In speaking of the meters, the author says:

"It is simply criminal to use a galvanic current without adequate means of knowing the amount actually passing through the patient."

We cannot agree with this statement, and the author's own words will sustain us in this opinion. It is certainly of scientific interest to measure exact amounts, but the sensations of the patient furnish the real indicator and guide to the current strength. The meter might easily and safely be dispensed with in careful hands, and just as good results be obtained. On page 61, the author himself says:

"Having reached the current strength desired in the case, *or the lesser amount that seems to be the limit of easy tolerance*, etc.

Again, page 65:

"The appearance of real pain should always be accepted as a sign for current reduction, and should lead to an immediate cessation of the treatment . . ."

And still again:

"The guide to the strength required in a given case is gained from the sensations of the patient; . . . . . if we do not shock by a sudden turning on or off, there is no possible way in which the patient can be harmed."

This shows very clearly that the author himself depends on pain as his guide, and not the meter.

The electrodes are described in the chapters devoted to each operation. Many of these are of the author's own invention, and overcome many of the defects of the old instruments. The experiments in the third chapter are briefly given, and well worth perusal.

The fourth chapter is devoted to the action of concentrated milliampère currents on organized tissues. Here we have the subject of the chemical polar and interpolar action fully discussed, and all the old arguments are used to sustain the now familiar position taken. We must, first of all, take exception to the clause—

"An experiment with fresh butcher's meat will give a very good illustration of the chemical part of these phenomena as they occur within the human body."

Experiments on dead tissues are always liable to lead one into error, and cannot stand as evidence alone and unsupported. We are willing to admit that the negative pole is alkaline and the positive is acid, and that at the one pole we obtain the soft liquefaction of an alkaline caustic, while at the other we observe the hardened, coagulated eschar of an acid caustic; but that there is any appreciable interpolar action, we must still consider unproven.

"The acids, oxygen, etc., appear at the positive pole, and the bases and hydrogen at the negative pole. An actual transfer of particles in both directions takes place through the whole distance of tissue between the poles, *but the freed particles, singularly enough, appear only in the immediate neighborhood of the latter.*"

This latter part of the clause is not only singular, but settles the whole question. The experiment given to sustain this absurd position, proves absolutely nothing. No change, either macroscopical or microscopical, has ever been satisfactorily demonstrated to have taken place interpolar; and until we have something more definite to judge from, we cannot accept it as true. The whole theory has apparently been bolstered up for the purpose of explaining some results of treatment, and there is no truth in it.

Two chapters are devoted to intra-uterine galvano-chemical cauterizations and pelvic electro-puncture Here we find some valuable hints as to the detail of instruments, their care and uses. The author objects to the sounds used by Apostoli and others, for the reason that they are covered by adjustable sheaths of such material that they cannot be subjected to a spirit flame, and because it is impossible to keep the interior of the tube clean. He has remedied these defects in an instrument he has invented, that is covered with gum-shellac. It can be easily cleaned with a flame, and renewed at will. The abdominal pad used is of clay, after the manner of Apostoli. For strengths under 100 milliampères, he uses a pad of absorbent cotton. To reach all parts of the endometrium during an operation, he continually moves the sound from side to side, instead of following the method of Apostoli and filling the cavity with a gelatinoid material. For years Apostoli carried on his treatment successfully, without an attempt to reach every point on the endometrium, and we cannot believe that this is essential to the treatment. At any rate, the kind of instrument used by the author could never be made to cauterize all parts. Twenty-four hours' rest in bed, after severe operations, is very wisely advised.

"With an even, unbroken current, . . . . . as much as three hundred milliampères may be given *without actual pain of any kind.*"

Why cannot men tell the truth about such matters? We have failed absolutely to find a single operator who uses electricity in large currents who does not get pain, and great pain. Even in the clinic of Apostoli himself, we have failed to see even one woman who did not complain bitterly of pain. As a rule, few women will allow over 100 milliampères to be administered to them without complaining loudly. We have repeatedly noted 50 and 60 milliampères as the utmost limit the operator dared employ, on account of pain. Science demands truth sooner or later. Why not admit it at once? The very quotations we have already used to show that the meter is not necessary, prove conclusively that the author himself obtains this same degree of pain in his own patients.

The author condemns all forms of puncture, except through the cervix or vagina, and then only to the depth of half an inch. He had done a great service to womankind had he condemned the puncture altogether. The procedure is not only unnecessary, but dangerous in the extreme. He has invented a new trocar, or fibroid spear, which, if such must be used at all, has many points in its favor.

One chapter is devoted to the faradic current, and we can best dispose of it by the following quotation:

"This current acts only on nerves and muscles, stimulating each into action, and its use is therefore limited strictly to such conditions as exhibit nervous and muscular laxity."

The franklinic current is used only in two conditions: "*backache of nervous women, and hysterical pains.*" For non-caustic vaginal, urethral and rectal applications, when currents over fifteen or twenty milliampères are used, they must be made with electrodes presenting large conducting surfaces.

The final chapter of the first part is given to a description of general galvanism and faradism.

The second part of the book is devoted to the treatment of the different diseases, and here, after all, will center the interest of the profession. This portion of the book contains ninety-nine pages, including an all-too-short a chapter on contra-indications, together with an appendix. The appendix gives directions for making battery fluids, amalgamating zincs, covering electrode discs, and making a cheap galvanic battery.

It is in this second part of the book that the author becomes the enthusiast, and, like all such, shows a disposition to claim any and everything in the most reckless manner. The time for the usefulness of the gynecologist (other than as an electrician) is past, as witness the list of diseases for which the only proper treatment is electricity: fibroid tumors and polypi, uterine hemorrhage (menorrhagia and metrorrhagia), endometritis, endocervicitis, subinvolution, uterine hyperplasia, pelvic indurations, acute and chronic inflammatory diseases of the pelvis, engorgements, displacements (versions, flexions and prolapses), foreign growths, obstructive dysmenorrhea, stenosis, inter-menstrual pelvic neuralgia, hysteria, extra-uterine pregnancy, amenorrhea, hydrosalpinx, and others. What a paralax this is of the words of the author (p. 193), in which he lectures against the "*indiscriminate employment of the powerful agency treated of in these pages.*" The only two diseases exempted from the electrical treatment, are papilloma of the broad ligament, of which the author has only had one case (and yet he bases his opinion wholly on this), and ovarian tumors, which he yet hopes to bring into the fold. Strange to say, it is recommended that pregnancy be avoided and left to nature. One might almost imagine, in reading these pages, that he were in fairy-land, or that he had inadvertently become engrossed in an advertisement in one of the daily papers.

Individual chapters teem with long-drawn-out details of the treatment of cases, and of course, like all such, represent the very best results obtained by the operator. A careful perusal of these picked cases show, many of them, as anything but satisfactory in their results. Numbers simply show a slight improvement, and are still "*under treatment.*" Some have passed through dangers such as most of us would hesitate to subject our patients to, and undoubtedly many others relapsed and are now in the hands of a physician for proper treatment. On page 69 we find: "*A most accurate diagnosis of the position of the tumor in relation to the important organs surrounding it, is first made,*" and the necessity of this accuracy of diagnosis is elsewhere reiterated. Now, an accurate diagnosis is oftener impossible than possible, as we all know full well, and if this is absolutely necessary for safe treatment, the remedy might as well be abandoned. But, fortunately, this is not always so; if electricity be limited to such cases as are suitable, this difficulty will be obviated to a great extent.

The great and successful field for this remedy is in the treatment of fibroid tumors. This chapter, to which forty-three pages have been devoted, finds the author at his best, and with the exception of a few points, on which we will comment, he is justified in what he recommends. He quotes at length from the Glasgow address of Apostoli, to sustain his position. Here Apostoli claims for his treatment:

1. An anatomical diminution which does not advance so far as the complete dispersal.
2. The quick and lasting cessation of hemorrhages.
3. The disappearance of all the symptoms of compression.
4. The symptomatic restoration of the patient.

Now, although we believe we have seen great good result from the use of large currents in fibroid tumors, yet there are many and great dangers possible, against which too much care cannot be taken.

On page 114, Apostoli is quoted:

"In certain women, with a large uterus and expanded cavity (*sic*), in which the ordinary sound had moved with great freedom, I have discovered, one, two, or three years afterward, that it could not be introduced, and that the canal only permitted the entrance of a sound of the most diminutive size."

Who is to say what shall be the limit of this cicatricial contraction, and are we not all well aware of the trouble caused by cicatrices in these parts? The author, apparently overlooking this fact, or perhaps thinking it could be frightened away by some magic influence, thinks, on page 166, "that in the positive cauterization we have the safest and most promising means of creating a *permanently patulous canal*, and a procedure that is destined to take the place of forcible dilatations."

What logic is this? Again, is there no danger from puncture? The author very wisely repudiates abdominal puncture, and he had done well had he thrown aside puncture altogether. His objection to abdominal puncture is just as applicable to the vaginal; in both, the trocar traverses the peritoneal cavity, and there is exactly as much danger of wounding an intestine below as above. With his usual consistency, the author, however, denies both these propositions. The only advantage obtained from the lower puncture, and it is a vital one, is good drainage. Both are uncertain and dangerous. Away with them! Puncture always means sloughing, fever, and the dangers of septicemia. Who is not familiar with the risks attending the opening of the capsule of a fibroid in enucleation from the uterine cavity, and the frequent stopping short of complete removal on that account? The dangers of puncture are quite the same. The electrode, recommended by the author, could not be worse. If the puncture is made, the whole length of the wound should be cauterized, so that it may not heal at the entrance and cause a suppurating retention cyst. The sphacelation and subsequent sloughing out of a tumor is looked upon as a triumph, and a case reported by a Dr. Holland is quoted. This case ran such a gauntlet of danger, that it is surprising to find it referred to as anything short of a disaster. We personally saw just such another case presented in triumph to the New York Obstetrical Society. The tumor had sloughed out through the posterior cul-de-sac. Both of these cases lived, but any man who would aim at such a method of termination, would act little short of criminally. These women can spend the rest of their days giving thanks that they are cured both of their disease and their doctors. And just here it might be well to know how many are not so fortunate as these favored few! We are cognizant of quite a large number of unreported deaths that have occurred in this country. In fact, our author's own city is rather prolific of just such cases. Surely he, of all others, cannot be in ignorance of what is commonly known. He mentions Cutter's list of fifty cases, with four deaths and only eleven cures, but tries to explain it away by assuming that Cutter and Kimball had not proper apparatus, and were not familiar with Ohm's law. It appears to us that Cutter and Kimball *had* worked in this field for years, even before the author entered it, and are amongst our pioneers and best of electricians. If we cannot accept their results, surely our author cannot ask us to accept his own. The quickness and completeness with which fibroid polyps are successfully removed surgically, contrasts markedly with the slowness and uncertainty of the electrical treatment, and leaves not the slightest room for doubt as to their proper management.

Chapter XII. deals with hemorrhages from the uterus from different causes. The course of treatment extends over two, three, and four weeks, and the relief is just such as we all see from a proper medical treatment, and in about the same time. Not a single point of advantage is presented. On the contrary, one of the picked cases was due to "retained secundines" following abortion, and the treatment extended over six weeks. The proper use of the curette would have cured such a case in a few days. If we are to use electricity for retained secundines, we will run somewhat the risk of leaving the path of enthusiasm to enter upon that of fanaticism.

Chronic Endometritis and Chronic Endocervicitis are dealt with in the next order, and separate and distinct lines of treatment are to be followed in each. Suffice it to say, that *gynecologists* have long since given up any such distinction between these diseases. In other words, they are one and the same disease, and never occur separately, and any treatment applied with any other idea in view is too unscientific to be even considered. "Here a destruction of the diseased layers must precede their regeneration," etc. Is it not much more reasonable to remove these diseased layers with a curette, and apply some mild stimulant, if you will, to the clean healthy surface, than to apply a strong current, leave dead tissue to come away by a slow process, and run the risk of cicatricial stenosis, to which we have already called attention? The contrast will not speak very well for electricity.

In Chapter XIV., the author states, "whenever chronic engorgement exists, a shrinkage to the normal size is an invariable record in these notes, and adhesions as well as exudations are generally noted as disappearing. . . . . The single contra-indication is the existence of pus." In making pus a contra-indication, the author does not follow Apostoli, who uses electro-puncture for its relief. The shrinkage spoken of is due to muscular contraction, which is in turn due to the stimulation of the uterine muscle by the electricity. Have we no other means to accomplish this same end? We undoubtedly have, and means that are just as reliable and as rapid; they also have the advantage of simplicity and easy use. Why should we throw away our old, tried, and valuable friends—ergot, hot-water injections, the tampon and others—for a remedy which is infinitely more troublesome and of no greater promise?

As to the disappearance of adhesions, we take the liberty of doubting that the author bases that statement on his own experience upon other than *a priori* grounds. There has never been, we asseverate, a single well-authenticated case put on record, in which the operator had succeeded in dissolving these adhesions. We have seen it attempted, time after time, only to end in miserable failures, even after six or seven months' trial. These organized adhesions of chronic pelvic inflammatory diseases are, to all practical purposes, part and parcel of the surrounding tissues. In fact, at times, when trying to break them up with a finger in the abdomen, the surrounding tissues often give way first. If they are melted away by this mysterious agent, so will be everything else in their neighborhood.

The next chapter is devoted to pelvic pain, and the author thinks "its abeyance or removal without the use of narcotics is usually a certain indication of restored health." Further, "it has not been unusual for patients who had exhibited the physiognomy of pain for years, to return smilingly to the clinic after but two or three applications." Precisely this relief from pain can be obtained from one free purgation, as we have ourselves noted time and time again. Case after case has come back to us after a free purgation, and refused operative aid, where before they had been anxious for it. But to assume that their relief means restored health, is absurd. The disease is still there, and the symptoms will return in full force as soon as treatment is discontinued, or something occurs to excite them to activity.

"Acute inflammatory affections, particularly perimetritis, are an absolute contra-indication to the use of strong currents. My own views coincide fully with those of Apostoli on this point." The author seems not to be familiar with some of Apostoli's recent writings, where he advocates just this use of electricity under certain precautions.

Under the heading, Nervous Dysmenorrhea, this author speaks of "an unnecessary shocking of wholesome modesty" of young girls by surgeons. It is now well recognized that most of the uterine complaints of this class of patients are referable more to the nervous system than to the pelvis, and there are few gynecologists who find it necessary to make more than a rectal examination, if they even go that far. With characteristic forgetfulness, our author himself details the case of a single lady, 21 years old, suffering from that (normal) condition of the uterus, ante-displacement. How did he know it was displaced? His other remarks, under the heads of Nervous Dysmenorrhea, Intermenstrual Pelvic Neuralgia, and Pelvic Pain of Hysteria, are quite timely, and well worthy of serious attention especially by some of the younger surgeons. We quite agree with the author that these cases for the most part belong to the neurologist.

Our author next treats of displacements of the uterus. The only case reported, as treated, is one of anteflexion, with a cavity measuring two and a half inches, plus. This poor woman, with a perfectly normal condition of her uterus, was treated for over six weeks. We all know how extremely difficult it is to treat cases of prolapse successfully, even with all our modern operations for suspending the uterus, and giving support from below. It can only be mentioned as an example of the extreme views of the author, that he cures prolapse with electricity.

A short chapter is devoted to the electrical treatment of extra-uterine pregnancy. Here we find that "the consensus of *intelligent opinion* in America inclines at the present time" to the use of electricity, for the purpose of killing the fetus before the fourth month. The justifiability of this treatment rests entirely on the ability of making a correct diagnosis:

even after the fetus is killed, the dangers are great. This the author admits, and then makes the following most remarkable statement :

"After the vitality of the ovum has been arrested, two courses are open to the surgeon : *either laparatomy for the removal of the dead mass, which is now more safely performed, etc.*"

Of this it is only necessary to say, that it is just as safe to remove the living as the dead mass, and any man who lacks the necessary pluck and skill, has no business to try that or any other abdominal operation. If electricians are going to advocate laparatomy to remove the *dead mass*, the profession will no longer pay any attention to their inconsistent protests against also removing the *living mass.*

To make a diagnosis, is extremely difficult, and the very men who are crying most loudly that they can make it have failed, as witness the cases of Mann, of this city, Kelly, of Philadelphia, and Buchmaster, of Brooklyn. Mann makes a diagnosis of extra-uterine pregnancy, and treats the fetus to a few doses of electricity. Wylie operates on the same case later, and removes a hydrosalpinx. Kelly operates on a case of extra-uterine pregnancy, and then tries to build up a post diagnosis. In the same report, he speaks of another case he has on hand, and states that he is *waiting for the fetus to die* before operating. The case turned out to be something else, and was never reported. Buchmaster applies electro-juncture to a pelvic tumor for some time, and finally announces to the patient that she is seven months pregnant. How are the mass of us to make a diagnosis when the immortals fail ? That the consensus of intelligent opinion of America turns to electricity in this disease, is hardly true. Surely the author cannot be ignorant of the fact that the vast—nay, almost whole—weight of opinion, in his own city, is most vigorously against it, and if he will look around him he will find it not quite so strong in other cities as he seems to imagine. New York, for instance, is about evenly balanced on this subject, with a leaning toward laparatomy.

Chapter XVIII. deals with Amenorrhea and Hydrosalpinx. In the first of these affections, there is room for electricity, and yet many cases of amenorrhea exist because the woman cannot afford to lose the blood, and, therefore, no local effort should be made to bring on the flow. Hydrosalpinx leaves absolutely no room for electricity, especially as it is impossible to diagnosticate it.

The last chapter is on Contra-indications, which consist of ovarian tumors and papilloma of the broad ligament. The only other contra-indications mentioned (Chap. V.) are:

1. During the menstrual flow.
2. If there is any acute metritis or peri-metritis.
3. If the woman is pregnant.

Under any other circumstances and with these exceptions, electricity can be used for, and cure, every ailment to which woman is liable.

The book is well written, clear and concise. The author's positions are firmly and positively taken, and will carry conviction to many minds. It is, however, filled with false teachings, and its very positiveness is its greatest fault, as it advances, as settled and accepted facts, some things that are as yet only in their infancy and on trial, and some others which have been found wanting and are dying out. Taken as a whole, it were much better that the book had never been written, as those who accept it as their infallible guide, are doomed to many disappointments, and to some such it may cause sad disasters. In consequence of all this, the reputation of electricity in those cases in which it is a most valuable therapeutic remedy, will suffer.

